# Effect of Yttrium Additions on the High-Temperature Oxidation Behavior of GH4169 Ni-Based Superalloy

**DOI:** 10.3390/ma17112733

**Published:** 2024-06-04

**Authors:** Tiantian Wang, Wei Liu, Shufeng Yang, Jingshe Li, Peng Zhao, Hui Xue

**Affiliations:** 1 School of Metallurgical and Ecological Engineering, University of Science and Technology Beijing, Beijing 100083, China; wangtiantian@xs.ustb.edu.cn (T.W.); lijingshe@ustb.edu.cn (J.L.); me_zhaopeng@163.com (P.Z.); xuehuiustb@xs.ustb.edu.cn (H.X.); 2 State Key Laboratory of Advanced Metallurgy, University of Science and Technology Beijing, Beijing 100083, China

**Keywords:** Ni-based superalloy, high-temperature oxidation, reactive element effect, yttrium, scale adhesion

## Abstract

The effect of the active element yttrium and its content on the oxidation behavior of GH4169 Ni-based superalloy at extreme temperature was studied by isothermal oxidation experiments. The results show that the oxide scale of GH4169 alloy presents a multi-layer structure, in which the continuous and dense Cr_2_O_3_ oxide layer is located in the subouter layer (II layer) and the continuous Nb-rich layer is in the subinner layer (III layer). These layers can inhibit the diffusion of oxygen and alloying elements, preventing the further oxidation of the alloy. The appropriate addition of yttrium can promote the selective oxidation of Cr element, reduce the thickness of the oxide scale and the oxidation rate of the alloy, inhibit the formation of voids at the interface of the oxide scale/alloy matrix, improve the resistance of the alloy to spalling as well as the adhesion of the oxide scale, and improve the high-temperature oxidation resistance of the alloy. Of those tested, the alloy containing 0.04 wt.%Y has the lowest oxidation weight gain, the slowest oxidation rate, and less oxide scale spalling. Based on this, the effect of yttrium on the high-temperature oxidation behavior of GH4169 Ni-based superalloy and its mechanism were revealed.

## 1. Introduction

GH4169 Ni-based superalloy is widely used in critical hot-end components of aero-engines due to its excellent high-temperature strength, microstructure stability, oxidation resistance, and corrosion resistance [1,2,3]. The operating temperature range of GH4169 alloy is usually from −253 to 650 °C, but the actual temperature may be higher than this range [4,5]. Superalloys that have been in long-term service under high-temperature conditions need to withstand complex interactions between temperature, stress, and the environment, which will inevitably undergo high-temperature oxidation reactions and accelerate component failure [6,7]. Therefore, it is necessary to investigate the high-temperature oxidation properties of GH4169 Ni-based superalloy at extreme temperatures.

The oxidation process of superalloys depends on the activity and content of the key alloying elements in the alloy, which requires that the alloying elements are required to have a stronger affinity for oxygen than the matrix elements, while the diffusion coefficient of metal ions in the oxide scale is as small as possible [8,9,10]. Generally, the content of Cr element in GH4169 Ni-based superalloy exceeds 15 wt.%, and the content of Al is less than 2–3 wt.%. Cr preferentially reacts with O to generate a structurally dense Cr_2_O_3_ oxide scale, which avoids further oxidation of the superalloy [11]. Studies have shown [12,13,14] that the oxidation resistance of superalloys is related to the formation of continuous Al_2_O_3_ or Cr_2_O_3_ oxide layers. The relatively dense structure of these oxide layers can significantly reduce the diffusion rate of various elements, thus reducing the oxidation rate. However, when the temperature exceeds 900 °C, Cr_2_O_3_ is unstable and prone to form volatile CrO_3_, which continuously consumes Cr in the matrix and thins the oxide layer [15]. In addition, if the service temperature of the alloy is too high, the cracking caused by the rapid growth of the oxide scale and the spalling of the oxide scale will invalidate the protective oxide scale and reduce the oxidation resistance of the superalloy [16,17]. Therefore, improving the spalling resistance and volatilization resistance of the Cr_2_O_3_ scale is critical to improving the oxidation resistance of GH4169 Ni-based superalloys at high temperatures.

The addition of active elements, such as Y, La, Hf, Ce, etc. [18,19], to the superalloy can promote the selective oxidation of Cr and Al to form a continuous dense oxide scale, significantly reduce the oxidation rate of the superalloy, as well as substantially improve the adhesion of the oxide scale to the alloy matrix. This phenomenon is known as the reactive element effect (REE) [14]. Li et al. [20] found that the addition of 0.05 wt.% of yttrium reduced the oxidation rate of Ni-16Mo-7Cr-4Fe alloy in the steady state stage by about 30 times, greatly promoted the selective oxidation of Cr element to form a dense Cr-O internal oxidation layer, and inhibited the outward diffusion of oxidizable elements, especially the volatile oxide-forming element Mo. The adhesion between oxide scale and matrix was significantly improved. Previous studies [14,21] have shown that yttrium is more active than the Cr element, and the Y_2_O_3_ oxide formed by the oxidation of yttrium can act as a heterogeneous nucleation core of Cr_2_O_3_ and promote the generation of protective oxide scale. Moreover, Yu et al. [22] conducted a cyclic oxidation test on K38 casting alloy with added yttrium. It was found that yttrium could not only promote the formation of the outer oxide scale and slow down the growth rate of the oxide scale, but also inhibit the occurrence of internal oxidation and nitridation, enhance the bonding of the oxide scale with the alloy matrix, and improve the anti-spalling performance of the oxide scale. Similarly, Weng et al. [21], Rehman et al. [23], and Shi et al. [24] found that yttrium can enhance the adhesion between the oxide scale and the alloy matrix and reduce the tendency for spallation of the oxide scale.

As GH4169 Ni-based superalloy is a key material for the preparation of aero-engine hot end components, the improvement of its oxidation performance is crucial, and how to give full play to the active element effect of rare earths is the focus of the current research. At present, there are few studies on the high-temperature oxidation properties of GH4169 alloy alloyed with rare earth, and the oxidation temperature is mainly below 950 °C [25,26,27,28]. Therefore, it is necessary to investigate the effect of yttrium on the oxidation behavior of GH4169 Ni-based superalloy at extreme temperature (1000 °C) and its oxidation mechanism. Based on this, GH4169 Ni-based superalloys with different additions of yttrium were prepared for high-temperature oxidation experiments in this study. The effects of yttrium and its content on the oxidation behavior of the superalloys and the oxidation mechanism were investigated through the methods of oxidation kinetic analysis, oxide phase analysis, surface morphology and cross-sectional morphology observation, and elemental distribution. It provides a reference for improving the high-temperature oxidation resistance of GH4169 Ni-based superalloy.

## 2. Materials and Methods

In this study, GH4169 Ni-based superalloy is melted by a triple melting process (vacuum induction melting + electroslag remelting + vacuum arc remelting, VIM + ESR + VAR), followed by solution and aging (960 °C, 1 h, air cooling → 720 °C, 8 h, furnace cooling at a rate of 50 °C/h → 620 °C, 8 h, air cooling). The chemical composition (wt.%) of the ingot is 0.028 C, 18.19 Cr, 0.072 Si, 0.24 Co, 0.065 Mn, 2.98 Mo, 5.42 Nb, 0.64 Al, 1.02 Ti, 17.11 Fe, and the balance of Ni, determined by inductively coupled plasma atomic emission spectroscopy (ICP-AES). The method of adding yttrium is as follows: the yttrium particles are wrapped with nickel foil and fixed onto the charging bar with nickel wire, placed at the charging position of a 2 kg vacuum induction furnace of laboratory grade, and added after the superalloy is melted, held for 30 min, and then air-cooled with power off. The content of yttrium in the ingot after remelting is shown in Table 1.

Samples with size of 11 × 10 × 3 mm^3^ were cut from the ingots and polished with 240~2000 grit sandpaper, cleaned with acetone and ethanol, and then dried for the following oxidation experiment. The oxidation experiment was conducted in an atmosphere furnace, and the experimental atmosphere was air. The treated samples were placed in an alumina crucible which had been pre-fired to a constant weight, and then positioned in the atmosphere furnace after it had been heated up to 1000 °C. The samples were taken out and cooled down to room temperature after being held for 10 h, 20 h, 50 h, 75 h, 100 h, 150 h, and 200 h. The samples were weighed using a balance with an accuracy of 0.01 mg and recorded.

The phase of the oxidation products on the surface of the samples was analyzed using X-ray diffraction (XRD, Rigaku SMARTLAB, Tokyo, Japan) with a scanning angle range from 10° to 90° and a scanning speed of 8°/min. The surface morphology of the samples after oxidation was investigated using scanning electron microscopy (SEM, ZEISS Gemini 300, Berlin, Germany) equipped with an energy dispersive spectrometer (EDS, INCA, Oxford, UK). The elemental distribution of the cross-sectional oxide scale was detected by an electron probe microanalyzer (EPMA, SHIMADZU EPMA- 1720H, Kyoto, Japan).

## 3. Results

### 3.1. Microstructure of the As-Cast Alloys

**Figure 1:**
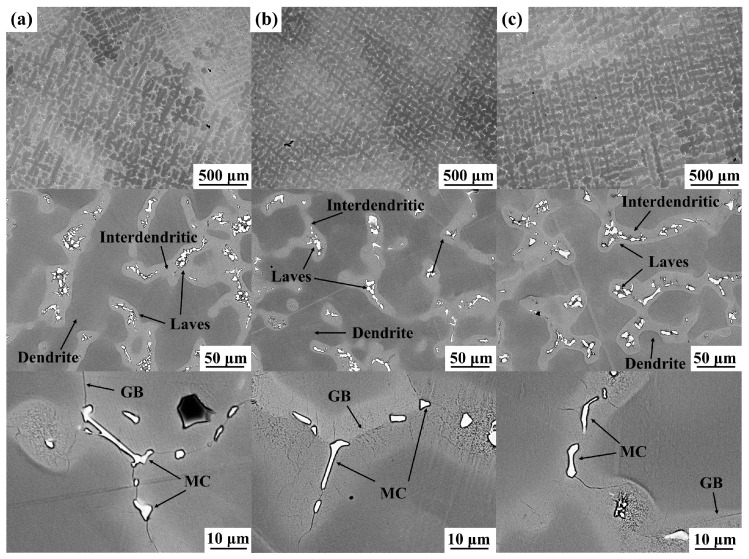
Microstructure of the as-cast GH4169 Ni-based superalloys: (**a**) Y0; (**b**) Y2; (**c**) Y4.

**Figure 2:**
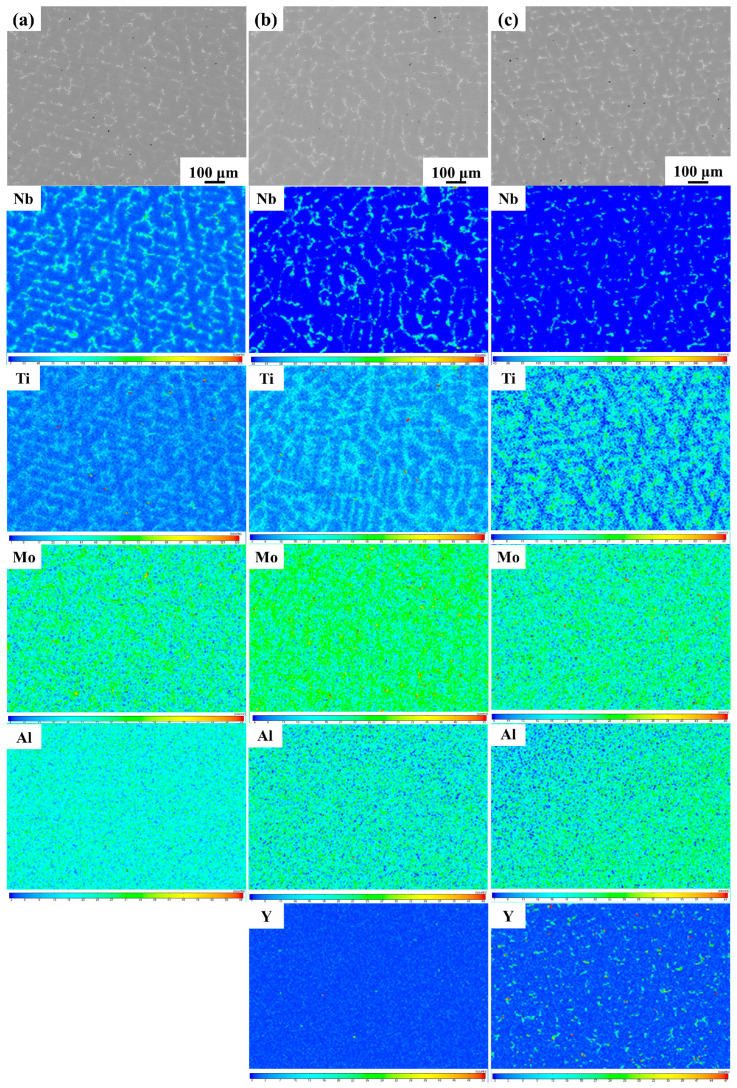
Distribution of elements of the as-cast GH4169 Ni-based superalloys: (**a**) Y0; (**b**) Y2; (**c**) Y4.

Figure 1 shows the microstructure of the as-cast GH4169 Ni-based superalloys Y0, Y2, and Y4 under backscattering. It can be seen that the alloys have similar microstructures and all have columnar dendrites. Among them, the dendrites of alloy Y2 are slightly refined and the dendrite spacing is reduced. The Laves phase and MC carbides in the alloys are mainly distributed in the interdendritic region and their compositions are shown in Table 2. The MC carbides are mainly composed of NbC and TiC. It can be seen from Table 2 that alloys Y2 and Y4 contain small amounts of Y in the matrix, Laves phase, and MC carbides. Figure 2 shows the distribution of the elements in the as-cast alloys. Alloy Y2 contains a small amount of yttrium, and there are more Y-rich particles in alloy Y2, which are mainly distributed in the dendrites. Studies have shown [20,29] that the solid solubility of yttrium in the superalloy is low, and when the content of yttrium exceeds the solid solubility, the Ni*_x_*Y*_y_* phase will be precipitated, which is not conducive to improving the oxidation performance of the alloy.

### 3.2. Oxidation Kinetics

**Figure 3:**
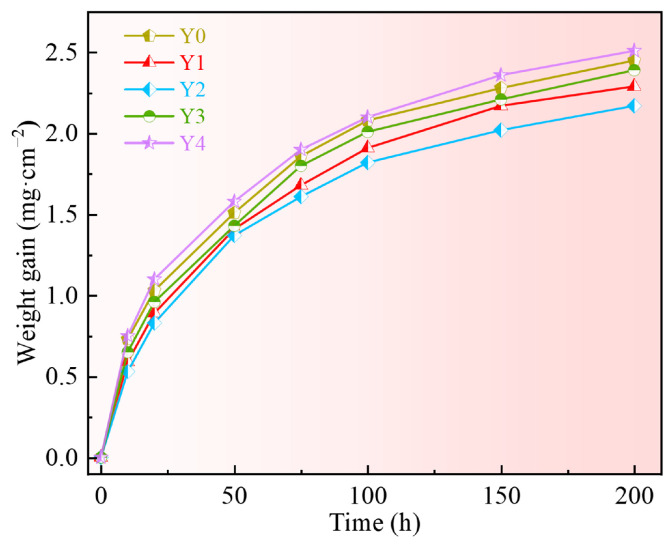
Oxidation kinetic curve of GH4169 Ni-based superalloy at 1000 °C.

The oxidation kinetic curves of GH4169 Ni-based superalloy with different additions of yttrium oxidized at 1000 °C for 200 h are shown in Figure 3. At the initial stage of oxidation, the surface of the superalloy is not protected by the oxide scale, and oxygen is in direct contact with the alloy matrix. This leads to faster reaction rates and oxidation weight gain, which is categorized as interface reaction control. As oxidation proceeds, the surface of the superalloy gradually formed a complete, dense oxide scale, reducing the direct contact between oxygen and the matrix. The oxidation reaction rate slowed down, and the oxidation reaction was transformed into elemental diffusion control. Based on the weight gain curves of the alloys over time, the oxidation weight gain of the five alloys is Y2 < Y1 < Y3 < Y0 < Y4. At the end of the entire oxidation process, the Y4 alloy gained the greatest amount of weight, and underwent more severe oxidation. The Y2 alloy, containing 0.04% Y, had the least weight gain and was less oxidized. The results indicate that adding yttrium appropriately has a beneficial effect on enhancing the high-temperature oxidation resistance of alloys, while excessive addition worsens the alloys’ oxidation.

### 3.3. Oxidation Products

**Figure 4:**
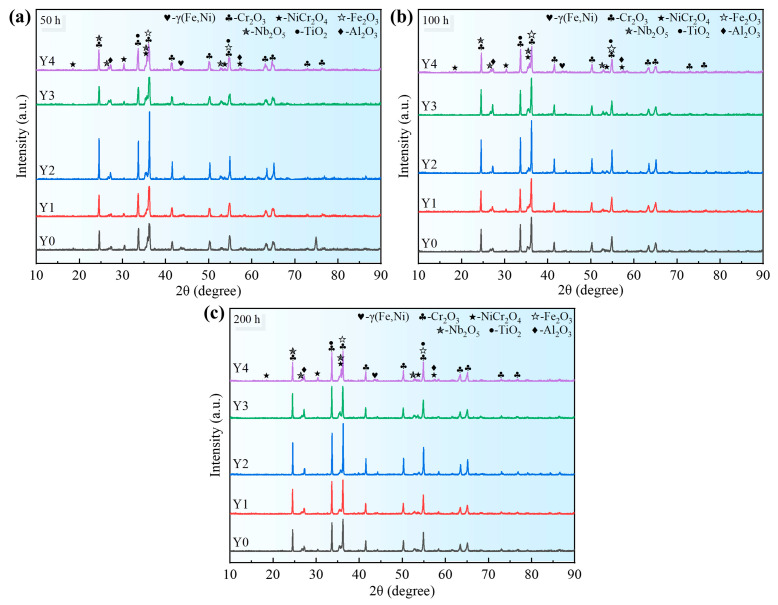
XRD patterns of GH4169 Ni-based superalloy with different oxidation times at 1000 °C. (**a**) 50 h; (**b**) 100 h; (**c**) 200 h.

Figure 4 shows the XRD patterns of GH4169 Ni-based superalloy with different additions of yttrium oxidized at 1000 °C for 50 h, 100 h, and 200 h, respectively. The oxide scale of the alloy with different yttrium was mainly composed of oxides such as Cr_2_O_3_, NiCr_2_O_4_, Fe_2_O_3_, Nb_2_O_5_, TiO_2_, and Al_2_O_3_. Additionally, the types of diffraction peaks of the oxides with different oxidation times are nearly consistent; only the relative intensities of the diffraction peaks are different. This indicates that the types of oxidation products of the alloys with different oxidation times are almost consistent and only differ in the content. During the above oxidation times, the relative intensity of the diffraction peaks of the matrix γ(Fe, Ni) phase is weaker, and the relative intensity of the diffraction peaks of Cr_2_O_3_ is stronger, indicating that the surface of the alloy is mostly covered by the Cr_2_O_3_ oxide scale.

The relative intensities of the Cr_2_O_3_ characteristic diffraction peaks of the Y2 alloy are significantly higher than those of the other alloys, indicating that its oxide scale has a higher content of Cr_2_O_3_. The dense Cr_2_O_3_ can effectively isolate the contact between the oxygen and the alloy matrix, inhibit the diffusion of oxygen atoms into the interior of the alloy matrix, and avoid further oxidation of the alloy [13].

### 3.4. Morphology of the Surface Oxide Layer

**Figure 5:**
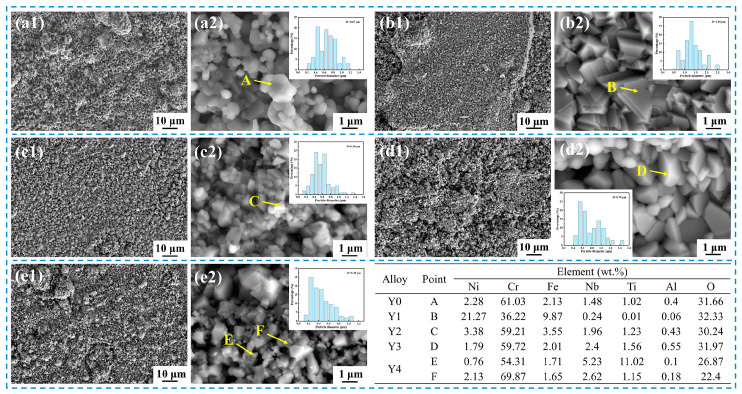
Surface morphology and EDS point analysis of GH4169 Ni-based superalloy after oxidation at 1000 °C for 50 h. (**a1**,**a2**) Y0; (**b1**,**b2**) Y1; (**c1**,**c2**) Y2; (**d1**,**d2**) Y3; (**e1**,**e2**) Y4.

The surface morphology of the oxide scale of GH4169 Ni-based superalloy oxidized at 1000 °C for 50 h is shown in Figure 5. The oxide scale of the alloy oxidized for 50h is relatively complete, and there is almost no spalling, but there are many raised oxide particle clusters. The surfaces of Y0, Y3, and Y4 alloys are uneven, indicating that the alloys are seriously oxidized. The surfaces of the alloys consist of numerous spherical and massive oxidized particles with uneven sizes. EDS analysis shows that these particles are mainly Cr-rich oxides (Cr > 55 wt.%), which can be judged to be Cr_2_O_3_ according to the analysis of the XRD pattern in Figure 4. The oxide particles on the surface of Y0 and Y3 alloys are relatively close to each other, and the size of oxide particles of Y4 alloy is not uniform. The EDS results show that there are some particles with higher Ti content (11.02 wt.%) on the surface of Y4 alloy. It may be due to the faster diffusion rate of Ti^4+^ in the oxide layer [30,31]. In contrast, the surface of Y1 and Y2 alloys is relatively flat, and the structure is relatively dense. According to the results of XRD and EDS, the oxide scale of Y1 alloy is mainly dominated by NiCr_2_O_4_ spinel with a regular shape, and the oxide scale of Y2 alloy is mainly composed of Cr-rich oxide (Cr_2_O_3_).

**Figure 6:**
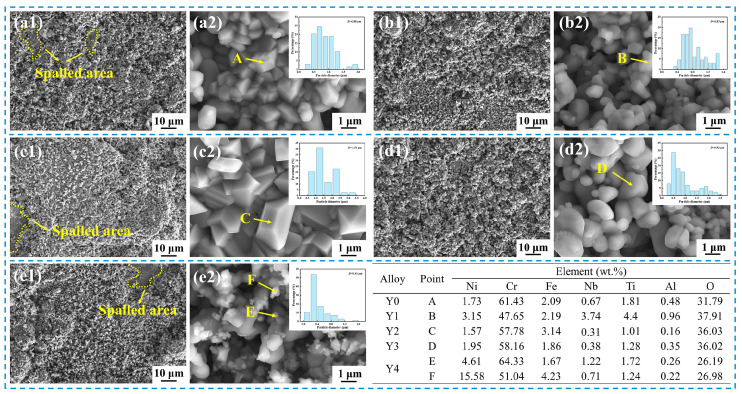
Surface morphology and EDS point analysis of GH4169 Ni-based superalloy after oxidation at 1000 °C for 100 h. (**a1**,**a2**) Y0; (**b1**,**b2**) Y1; (**c1**,**c2**) Y2; (**d1**,**d2**) Y3; (**e1**,**e2**) Y4.

The surface morphology of the oxide scale of GH4169 Ni-based superalloy oxidized at 1000 °C for 100 h is shown in Figure 6. As shown in Figure 6, the oxide scale of the alloy oxidized for 100 h appears slightly spalled, and the surface structure is looser and more porous. The size of the oxide particles has increased, and the oxide particles are stacked on top of each other. The composition of the oxide particles is basically the same as those of oxidized 50 h, mainly the Cr-rich oxides and Ni-Cr-rich oxides. Spalling of the oxide scale on the surface of the alloy may be caused by the stress generated during the growth of the alloy oxide scale or the mismatch between the thermal expansion coefficient of the oxide and the alloy matrix [32,33]. Once the oxide scale spalling occurs, the alloy matrix is exposed to the oxidizing environment again, which will further accelerate the oxidation of the alloy.

**Figure 7:**
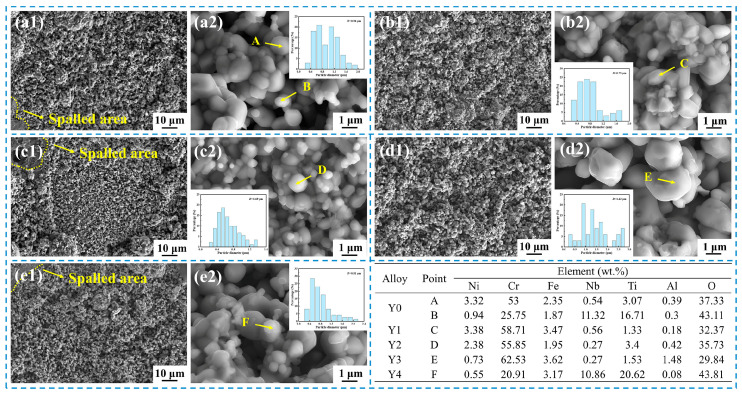
Surface morphology and EDS point analysis of GH4169 Ni-based superalloy after oxidation at 1000 °C for 200 h. (**a1**,**a2**) Y0; (**b1**,**b2**) Y1; (**c1**,**c2**) Y2; (**d1**,**d2**) Y3; (**e1**,**e2**) Y4.

Figure 7 shows the surface morphology of the surface oxide scale of GH4169 Ni-based superalloy oxidized at 1000 °C for 200 h. The surface of the alloy is almost entirely covered by raised clusters of oxide particle, the degree of looseness has further increased, and the size of the oxide particles has enlarged, as depicted in Figure 7. Mao et al. [34] believed that the metal cations in the alloy, during the process of diffusion outward, would react with the inward-diffusing O^2−^ from the air, generating new oxide particles. Consequently, the oxide scale would grow horizontally, increasing the total area and forming oxide scale wrinkles, gradually leading to the formation of a large oxide particle cluster. Simultaneously, the outward diffusion of cations leaves vacancies in the matrix, which accumulate at the interface of oxide scale/matrix to produce cavities, causing localized detachment of the oxide scale from the matrix. In addition, EDS results indicated that the contents of Ti (16.71–20.62 wt.%) and Nb (10.86–11.32 wt.%) in the oxide particles of Y0 and Y4 alloys increased, while the content of Cr (20.91–25.75 wt.%) decreased significantly. It may be due to the faster outward diffusion rates of the metal cations Ti^4+^ and Nb^5+^, which react with oxygen in the air to form Nb-Ti-rich oxide particles [30,31]. The loose structure of the surface oxide scale and the spalling of the oxide scale provide a channel for the internal diffusion of oxygen, which will accelerate the oxidation of the alloy.

### 3.5. Cross-Sectional Analysis

**Figure 8:**
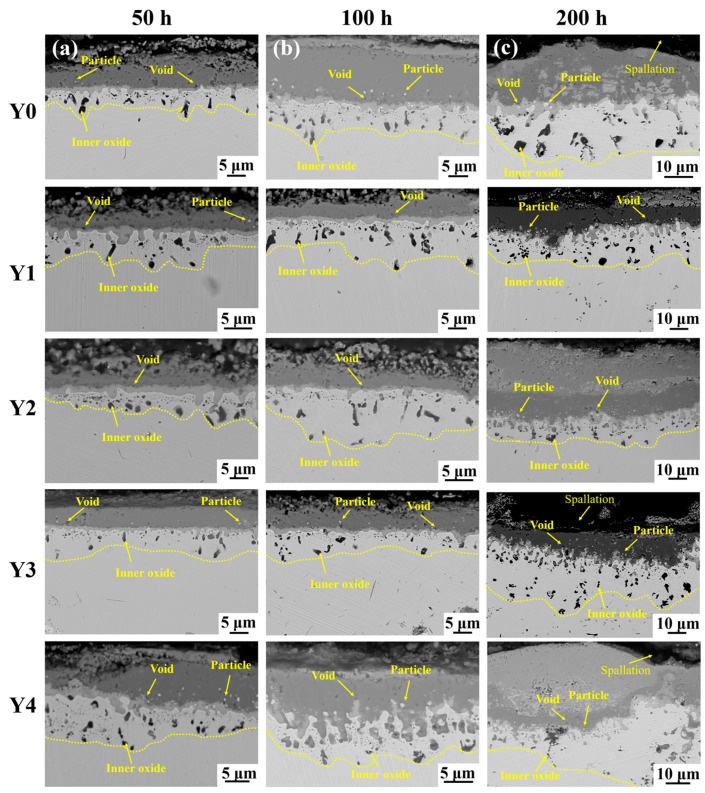
Cross-sectional morphology of GH4169 Ni-based superalloy oxidized at 1000 °C for different times. (**a**) 50 h; (**b**) 100 h; (**c**) 200 h.

Figure 8 shows the cross-sectional morphology of GH4169 Ni-based superalloy oxidized at 1000 °C for different times. It can be seen that the oxide scale of the alloy presents a multilayer structure after different oxidation periods. As the oxidation proceeds, the thickness of the oxide scale gradually increases, and part of the oxide scale is obviously spalled, and the internal oxides gradually increase. At 50 h of oxidation (Figure 8a), the thickness of the oxide scale is thin and flat, the number of oxides in the innermost layer is small, and there are voids and bright white particles at the interface of the oxide scale/alloy matrix. Among them, Y4 alloy has the largest amount of bright white particles at this interface. At 100 h of oxidation (Figure 8b), the thickness of the alloy oxide scale and the number of oxides in the innermost layer gradually increased. More bright white particles and voids appeared at the interface of the oxide scale/alloy matrix for Y0 alloy, followed by the number of bright white particles for Y4 alloy. The number of bright white particles for the other alloys was less. When oxidized for 200 h (Figure 8c), the structure of the outermost oxide layer of the alloy becomes more loose, and part of the oxide scale is noticeably spalled off, and the number of oxides in the innermost layer increases significantly. Particularly for alloy Y0, after oxidation for 200 h, the oxide scale thickened dramatically, the depth of the inner oxide layer increased, the structure of the oxide scale was more loose and porous, and the outermost oxide layer largely spalled off.

By comparing the oxide scale of the five alloys, it was found that the thickness of the oxide scale of Y0 alloy without the addition of yttrium was significantly higher than that of Y1, Y2, and Y3 alloys with the addition of yttrium for short-term oxidation (50 h and 100 h), but that the thickness of the oxide layer of Y4 alloy was higher than that of Y0. When oxidized for a long time (200 h), the thickness of the oxide layer of the alloy further increased, and the outermost oxide scale appeared to have spalled off to varying degrees. Among them, the outermost oxide scale of Y0 alloy was basically spalled off. The above phenomenon shows that the addition of an appropriate amount of yttrium can slow down the oxidation of the alloy, reduce the spalling of the oxide scale and improve the high-temperature oxidation resistance of the alloy. However, an excessive amount of yttrium will reduce its high-temperature oxidation resistance.

**Figure 9:**
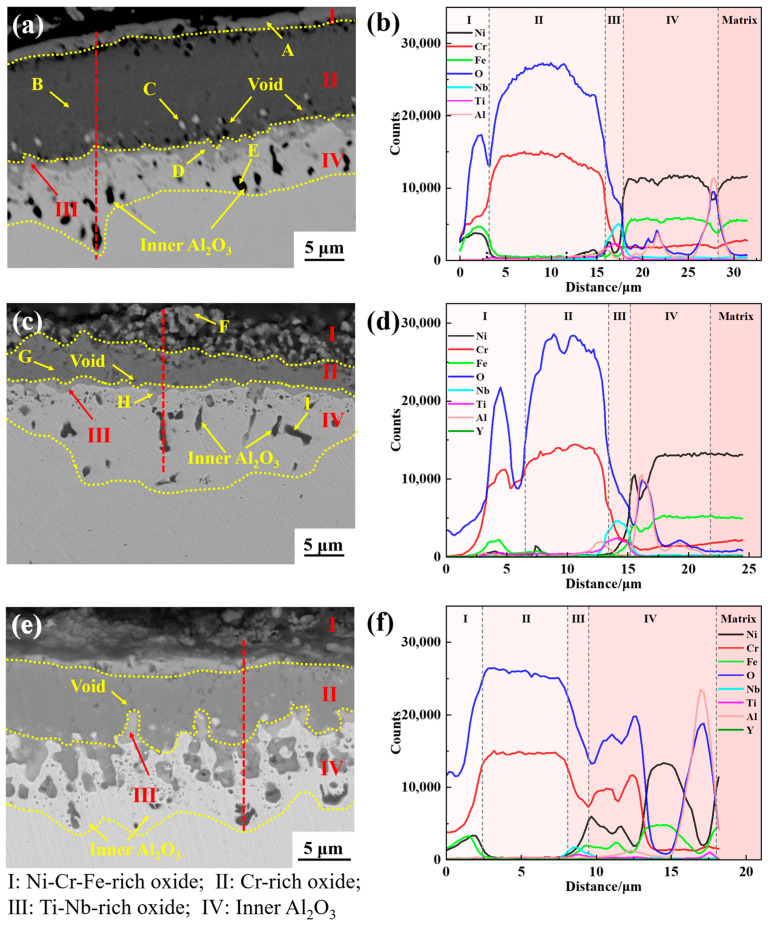
Cross-sectional elemental line distribution of the oxide scale of GH4169 Ni-based superalloy after oxidation at 1000 °C for 100 h. (**a**,**b**) Y0; (**c**,**d**) Y2; (**e**,**f**) Y4.

**Figure 10:**
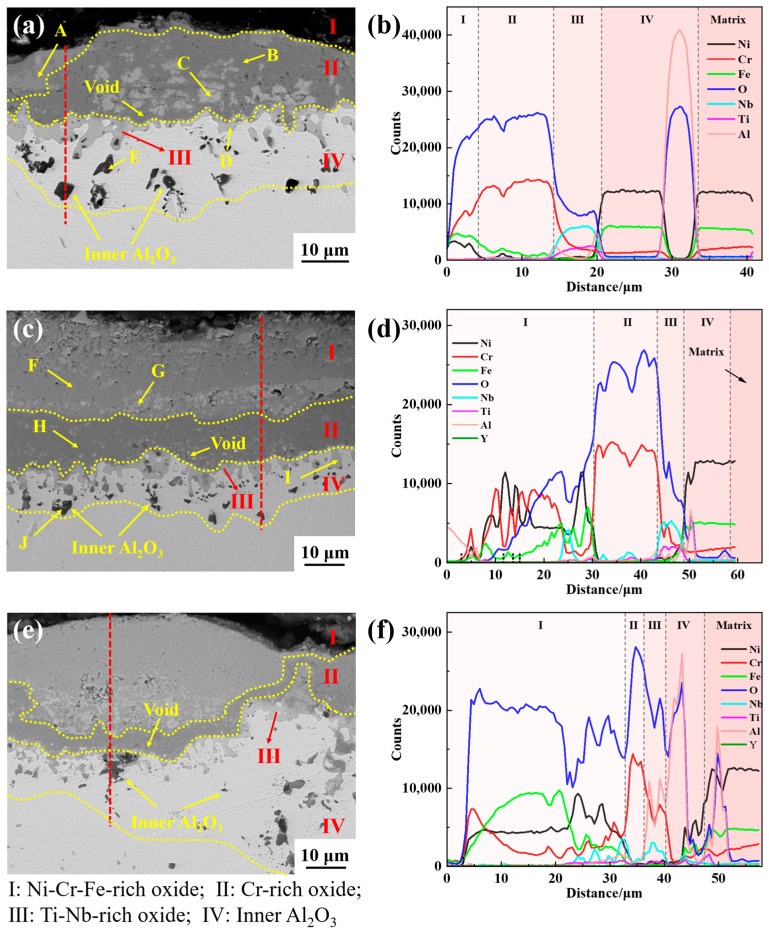
Cross-sectional elemental line distribution of the oxide scale of GH4169 Ni-based superalloy after oxidation at 1000 °C for 200 h. (**a**,**b**) Y0; (**c**,**d**) Y2; (**e**,**f**) Y4.

Taking Y0, Y2, and Y4 alloys as examples, the element distribution in the cross-sectional oxide scale of GH4169 Ni-based superalloy was analyzed, as shown in Figure 9 and Figure 10. For the alloy oxidation of 100 h and 200 h, the oxide scale is mainly divided into four layers, the outermost layer (I layer), subouter layer (II layer), subinner layer (III layer), and innermost layer (IV layer). The structure of the I layer is relatively loose and mainly composed of Cr-Ni-Fe-O elements. According to the XRD results and EDS analysis in Table 3 and Table 4, it is inferred that the layer is mainly composed of Cr_2_O_3_, spinel NiCr_2_O_4_, and Fe_2_O_3_. The structure of II layer is dense, mainly rich in Cr-O, and its oxide is presumed to be Cr_2_O_3_. The subinner layer (III layer) is mainly rich in Nb-Ti-O, and the oxides of this layer are TiO_2_ and Nb_2_O_5_. The innermost layer (IV layer) is mainly composed of Al and O elements, and the main oxide is Al_2_O_3_.

After 100 h of oxidation, the thickness of the oxide layer on the Y0 alloy was significantly greater than that of the Y2 and Y4 alloys, as shown in Figure 9. The thicknesses of the oxide layer on the Y0, Y2, and Y4 alloys were roughly 17.57 μm, 9.28 μm, and 13.15 μm, respectively, and the depths of oxidation of the innermost layer were around 6.84 μm, 8.5 μm, and 5.62 μm, respectively. At the same time, there were more voids present at the interfaces of the outermost layer (I layer)/subouter layer (II layer) and the interfaces of the subouter layer (II layer)/subinner layer (III layer) had more voids. By contrast, the number of voids in Y2 and Y4 alloys is significantly lower than that of Y0 alloy, and the size is smaller. This shows, to a certain extent, that yttrium is capable of inhibiting the formation of voids. The formation of voids will not only lead to a reduction in the density of the oxide scale, providing a channel for the internal diffusion of oxygen and exacerbating the degree of internal oxidation of the alloy, but will also reduce the bonding strength of the oxide scale with the alloy matrix, resulting in the oxide scale being extremely susceptible to spalling [35].

Figure 10 shows the typical cross-sectional morphology and line scan results of Y0, Y2, and Y4 alloys oxidized for 200 h. As can be seen from the figure, the oxide scale of the alloys thickened dramatically, the depth of the innermost oxidized layer increased, and the structure of the oxide scale became more loose and porous. The outermost (I layer) oxide scale of Y0 alloy was basically spalled, and the remaining oxide layer with a thickness of about 20.91 μm was adhered to the surface of the alloy matrix, with the depth of the innermost oxidized layer about 18.2 μm. In contrast, Y2 alloy has the least oxide scale spalling, with the oxide layer thickness of about 39.97 μm and the depth of the innermost oxide layer of about 10.44 μm. Y4 alloy has obvious oxide scale spalling, with the thickness of the oxide layer of about 33.6 μm and the depth of the innermost oxide layer of about 19.78 μm. When oxidized for 200 h, the outermost oxide layer of Y0 alloy almost spalls off completely, and Y2 alloy containing 0.04% of yttrium has less spalling, which indicates that the addition of the appropriate amount of yttrium can improve the adhesion of the oxide scale and reduce the spalling of the oxide scale.

The bright white particles exist at the interface between the subouter layer (II layer) and the subinner layer (III layer) of the alloy, as shown at point C in Figure 9, and the EDS results in Table 3 indicate that the particles are NiCr_2_O_4_. The presence of NiCr_2_O_4_ particles will destroy the densification and continuity of the Cr_2_O_3_ oxide layer. When oxidized for 100 h, the highest number of bright white particles appear at the interface between the subouter layer (II layer) of Y0 alloy, followed by Y4 alloy. Y2 alloy has the least amount of particles.

Additionally, from the EDS results in Table 3 and Table 4, it can be observed that there is a distribution of yttrium in the subinner layer (III layer) of alloy Y2, despite the relatively small quantity. However, according to previous studies, yttrium can reduce the thickness of the alloy oxide layer to a certain extent, and reduce the number of voids at the interface between the oxide scale and the alloy matrix, thereby enhancing the adhesion between the oxide scale and the matrix. This is consistent with previous research findings [21,24].

**Figure 11:**
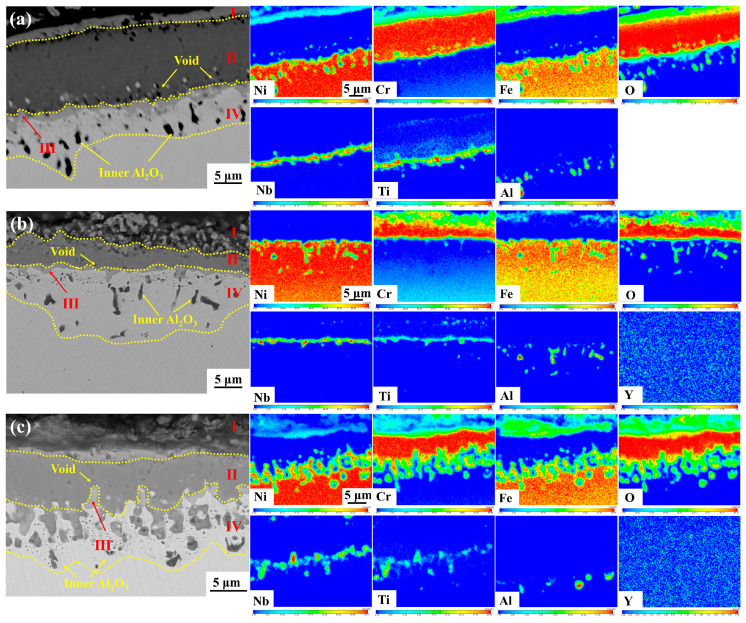
Cross-sectional morphology and elemental distribution of GH4169 Ni-based superalloy oxidized at 1000 °C for 100 h. (**a**) Y0; (**b**) Y2; (**c**) Y4.

**Figure 12:**
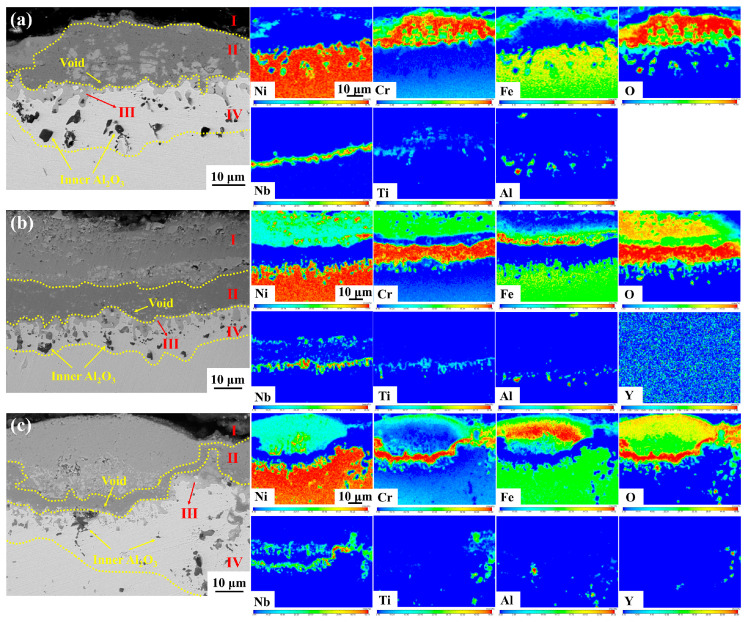
Cross-sectional morphology and elemental distribution of GH4169 Ni-based superalloy oxidized at 1000 °C for 200 h. (**a**) Y0; (**b**) Y2; (**c**) Y4.

Figure 11 and Figure 12 show the elemental distribution in the cross-section oxide scale of GH4169 Ni-based superalloy oxidized for 100 h and 200 h, respectively. It can be seen that the subinner layer (III layer) of the alloy is a continuously distributed Nb-rich oxide layer. It has been shown that TiO_2_ has a rutile structure at high temperatures, and at higher oxygen partial pressures, oxygen vacancies are its main defects, and TiO_2_ grows through oxygen diffusion in the oxide layer mainly via the vacancy mechanism [36]. Nb^5+^ can replace Ti^4+^ in TiO_2_, which is capable of reducing the concentration of oxygen vacancies and inhibiting the growth of TiO_2_. This results in the formation of a stable Nb-Ti-rich oxide layer, which is more protective than the TiO_2_ layer [37]. The continuous Nb-Ti-rich oxide layer can inhibit the further oxidation of the alloy and improve the high-temperature oxidation resistance of the alloy [38].

During oxidation, the metal ions Nb^5+^ and Ti^4+^ diffuse outwards while O^2−^ diffuses inwards. At shorter oxidation times, the Nb-rich oxide layer primarily appeared in the subinner layer (III layer). Following 200 h of oxidation, Nb^5+^ and Ti^4+^ further diffuse to the subouter layer (II layer) and the outermost oxide layer (I layer), forming a large area of discontinuous and unevenly distributed Nb-Ti-rich oxides in the subouter layer (II layer) of Y0 alloy (as illustrated in Figure 12), which destroys the dense protective oxide scale of Cr_2_O_3_ in the subouter layer (II layer), and reduces the oxidation resistance of the alloy.

The Nb-Ti-rich oxides appear in the outermost oxide layer (I layer) and subouter layer (II layer) of the oxide scale of Y2 and Y4 alloys, which are relatively uniformly distributed and small in size. Since the outermost layer of the oxide scale of Y0 alloy is almost completely spalled, it is impossible to compare the distribution of Nb-Ti-rich oxides in the outermost oxide layer (I layer) of its oxide scale.

## 4. Discussion

### 4.1. Effect of Yttrium on the Oxidation Rate

At the initial stage of oxidation, the oxidation process is controlled by the chemical reaction between the alloy matrix and oxygen, and the weight gain rate is fast. With the extension of oxidation time, a protective oxidation scale is formed on the alloy matrix surface, and the oxidation rate slows down. The oxidation kinetics can be preliminarily judged according to the oxidation weight gain reaction index (*n*). It is generally believed that the oxidation weight gain of alloys follows the following equations [39,40]:

(1) $${\left(\Delta W\right)}^{n}=K_{p}t$$

(2) $$\ln\left(\Delta W\right)=1/n\ln t+1/n\ln K_{p}$$

where ∆*W* is the oxidation weight gain per unit area, mg·cm^−2^; *t* is the oxidation time, h; *K_p_* is the oxidation rate constant, mg*^n^*·cm^−2*n*^·h^−1^.

**Figure 13:**
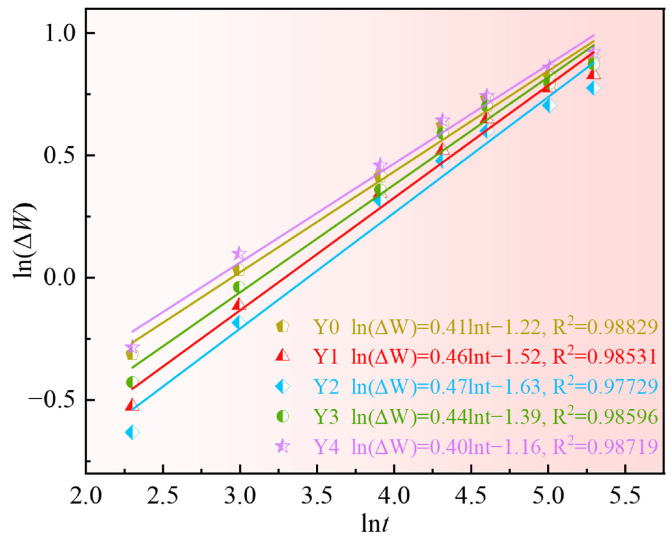
Logarithmic curve of oxidation weight gain of GH4169 Ni-based superalloy at 1000 °C.

The ln(∆*W*) and ln*t* data are plotted in Equation (2) and fitted by a linear regression, as shown in Figure 13. The resulting graph approximates a straight line law between the two, with the slope and intercept of the line representing 1/*n* and *K_p_*, respectively, as displayed in Table 5.

The oxidation rate constants of Y1, Y2, and Y3 alloys with added yttrium are all lower than that of Y0 alloy without yttrium, indicating that yttrium can reduce the oxidation rate of the alloys. However, the oxidation rate constant of Y4 alloy with 0.25% Y is higher than that of Y0, indicating that excessive yttrium will deteriorate the oxidation resistance of the alloy. In addition, the oxidation gain reaction index *n* of the five alloys with different additions of yttrium is between 2 and 3 in all cases, illustrating that the alloy oxidation weight gain curve is between the parabola and the cubic law. The oxidation process is controlled by the diffusion of ions in the oxide scale, and GH4169 superalloy has good oxidation resistance at high temperatures.

### 4.2. Effect of Yttrium on Scale Adhesion

In this study, the number of voids in the Cr_2_O_3_ oxide layer and at the oxide scale/matrix interface was significantly higher in Y0 alloy without yttrium than in Y2 alloy with yttrium, as shown in Figure 9 and Figure 10. In the process of high-temperature oxidation, a large number of cation vacancies (Kirkendall vacancies) are generated at the oxide scale/matrix interface due to oxidation and elemental diffusion, and these vacancies will continuously deposit and grow at the interface, and eventually form larger voids [26,41]. These voids will disrupt the continuity of the Cr-rich oxide layer, thus accelerating the inward diffusion of oxygen, while the voids will reduce the bonding strength between the oxide scale and the alloy matrix, making the oxide scale susceptible to spallation, and decreasing the protection and stability of the oxide scale. The active elements can inhibit the nucleation and growth of voids at the interface between the oxide scale and the alloy matrix. The oxides formed by the active elements in the alloy can be used as vacancy deposition sources to reduce the accumulation of vacancies and the formation of voids at the interface, thus improving the bonding strength of the oxide scale [35,42,43]. Similarly, previous studies [20,21,22,23,24] have shown that the active element yttrium enhances the adhesion between the oxide scale and the alloy substrate, and reduces the tendency of the oxide scale to spall.

The growth rate of the oxide scale is closely related to the external diffusion behavior of the active elements [44,45]. The active element ions have the same diffusion path as the metal cations. The active element yttrium ions have a larger radius and diffuse more slowly along the grain boundaries of the oxide scale, which can effectively inhibit the external diffusion process of metal cations and reduce the oxide scale growth rate [46]. Studies [34,47] have shown that yttrium can inhibit the outward diffusion of cations along the grain boundaries, so that the inward diffusion of O^2−^ along the grain boundaries of the oxide scale becomes a speed control factor for the growth of the oxide scale. The vacancy defects resulting in voids at the oxide scale/matrix interface are significantly reduced. Since no voids can be formed at the interface, the adhesion of the oxide scale is radically improved. This is consistent with the phenomenon observed in this study.

In general, the appropriate addition of active element yttrium can promote the selective oxidation of Cr element, reduce the thickness of the oxide scale and the oxidation rate of the alloy, inhibit the formation of voids at the interface of the oxide scale/matrix, and improve the alloy resistance to spalling and the adhesion of the oxide scale. However, excessive addition of active element yttrium will reduce the antioxidant performance of the alloy.

### 4.3. Oxidation Mechanism

**Figure 14:**
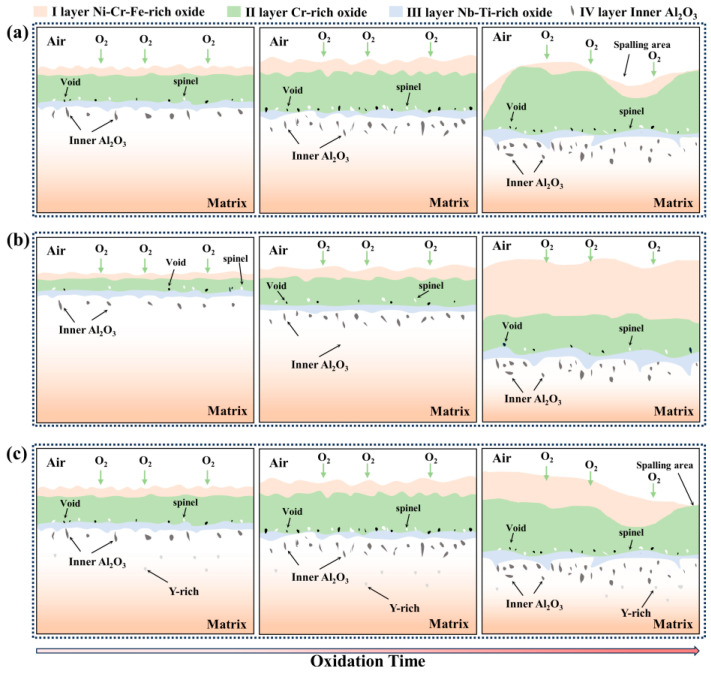
Schematic diagram of oxidation mechanism: (**a**) yttrium-free; (**b**) appropriate yttrium; (**c**) excessive yttrium.

Due to the affinity between the elements and oxygen, and the varying content of elements in the alloy, as well as differences in the elements’ diffusion ability, a multi-layer oxide scale structure is formed after the GH4169 Ni-based superalloy is oxidized at 1000 °C. The outermost layer (I layer) is mainly composed of Cr_2_O_3_, a small amount of NiCr_2_O_4_ spinel, and Fe_2_O_3_, etc. The subouter layer (II layer) mainly consists of dense Cr_2_O_3_ oxides. The subinner layer (III layer) consists of TiO_2_ and Nb_2_O_5_. The innermost layer (IV layer) is primarily Al_2_O_3_. This is mainly determined by the affinity between each element and oxygen, the element content, and the diffusion ability of the elements. The oxidation mechanism schematic diagram of GH4169 Ni-based superalloy is shown in Figure 14.

At the initial stage of oxidation, the oxygen in the air at high temperature contacts the alloy matrix, is adsorbed to the alloy surface, and reacts with the alloying elements to form oxide nuclei which grow and gradually form a complete thin oxide scale on the alloy surface, usually for a short period in the early stages of oxidation. Subsequently, diffusion of oxygen and metal ions through the oxide scale occurs, new nuclei are formed and grow, and the thickness of the oxide scale continues to increase. As oxidation proceeds, a continuous and dense oxide scale is formed on the surface of the alloy, which can effectively prevent contact between oxygen and the matrix. The oxidation process is controlled by the diffusion of ions in the oxide scale. During the oxidation process, the cationic vacancies generated by the diffusion of elements gradually form voids, which destroy the continuity and denseness of the oxide scale. The formed oxide scale grows, the thickness increases, and the Al_2_O_3_ oxide formed in the area of the inner oxide layer also increases gradually. With the further deepening of the degree of oxidation, oxide scale spalling occurs, and the spalling area is in contact with the air again to form a new oxide scale. The growth rate of the oxide scale, the formation of voids at the interface between the oxide scale and the alloy matrix, and the spalling of the scale can be reduced by the addition of an appropriate amount of active element yttrium. The addition of excessive yttrium in the alloy will form a Y-rich phase and reduce the high-temperature oxidation resistance of the alloy. The specific composition of the Y-rich phase formed by the excessive addition of yttrium is an issue to be explored in future research.

**Figure 15:**
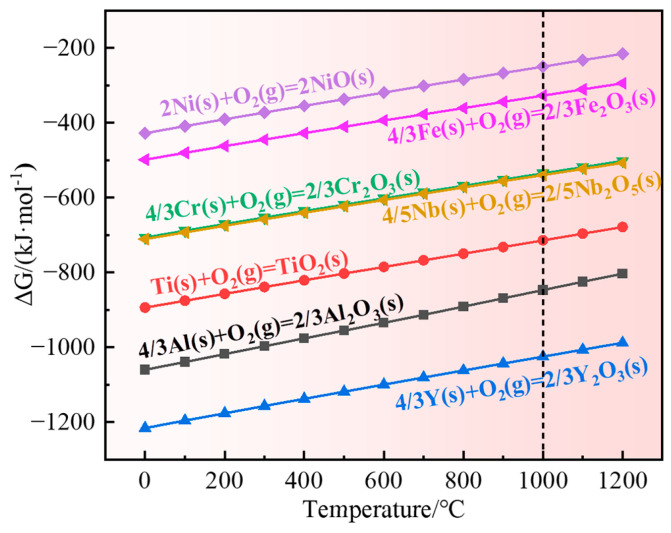
Gibbs free energy diagrams for oxides.

The Gibbs free energy diagrams for the reaction of 1 mol O_2_ with the elements in GH4169 Ni-based superalloy to form the corresponding oxides in the temperature range of 0–1200 °C were calculated using HSC 6.0 thermodynamic software as shown in Figure 15. The results of the Gibbs free energies for the formation of the respective oxides at 1000 °C are shown in Table 6. The Gibbs free energy of these possible oxides is less than 0, indicating that these oxides can be formed. Among these elements, yttrium has the largest oxygen affinity, and it is easy to form Y_2_O_3_ during the oxidation process, followed by Al, Ti, Nb, Cr, Fe, and Ni. The lower the Gibbs free energy of the reaction with O, the easier it is for the oxide to be formed. Nevertheless, the formation of the oxide scale is the outcome of the combination of thermodynamics and kinetics, which is related to the alloy’s element content and the elements’ diffusion ability.

Based on the Gibbs free energy calculations in Table 6, the Gibbs free energy of Cr_2_O_3_ formation is higher than that of Al_2_O_3_, but due to the higher content of Cr in the alloy (17~21 wt.%) than A1 (0.2~0.8 wt.%), the diffusion rate of Cr^3+^ is higher than that of Al^3+^ [44,45]. Therefore, the initial stage of oxidation is dominated by selective oxidation of Cr, and a large amount of Cr_2_O_3_ is rapidly formed covering the alloy surface. Meanwhile, Ni, as the matrix element of GH4169 Ni-based superalloy, has a content of more than 50%, which is much higher than other elements, and it is easy for it to react with O to produce NiO. NiO is extremely easy to grow and easy to spall off, and it has no protective effect on the alloy. As oxidation proceeds, NiO reacts with Cr_2_O_3_ to form NiCr_2_O_4_ spinel [48]. The content of Fe element in the alloy is slightly lower than that of Cr element, and it is easy for it to combine with oxygen to form Fe_2_O_3_ at high temperature. Consequently, the outermost layer consisting mainly of Cr_2_O_3_ with a small amount of NiCr_2_O_4_ and Fe_2_O_3_ oxides is gradually formed.

In addition, despite the low Ti content (0.65–1.15 wt.%) in the matrix, it is more active than Cr, and the diffusion rate of Ti^4+^ in the Cr_2_O_3_ oxide layer is greater than that of Cr^3+^ [30,31]. Therefore, it will diffuse between different oxide layers throughout the oxidation process and combine with oxygen to form TiO_2_ or titanium-containing oxides. However, the high Cr and low Ti contents in the alloy prevent the formation of a continuous TiO_2_ oxide layer on the surface of the outer oxide layer, but only a small amount of granular TiO_2_.

From the thermodynamic calculation in Figure 15, Nb has a high affinity for oxygen and is prone to form Nb_2_O_5_, which is consistent with previous studies [49,50]. Secondly, Nb can promote the formation of Cr_2_O_3_ protective layer by reducing the solubility of oxygen in the alloy [40,51]. Studies have shown [46] that the formation of Nb_2_O_5_ can act as a binding agent, enabling the formation of a more continuous oxide scale between the originally almost incompatible TiO_2_ and Al_2_O_3_. The continuous Nb-rich layer formed beneath the Cr_2_O_3_ protective layer could impede the outward diffusion of oxygen and metal elements and prevent additional oxidation of the alloy [21,38,52]. In this study, a continuous Nb-rich layer is formed beneath the Cr_2_O_3_ protective layer, which could impede the internal diffusion of oxygen and further oxidation of the alloy. In addition, it can be seen from Table 3 and Table 4 that a small amount of yttrium is also distributed in this layer, which has a high oxygen affinity and may oxidize to Y_2_O_3_. Y_2_O_3_, as the heterogeneous nucleation core of Cr_2_O_3_, promotes the formation of protective oxide scale, thus slowing down the outward diffusion of matrix elements and the inward penetration of oxygen, and reducing the oxidation rate [21]. In this study, yttrium may act in the form of Y_2_O_3_. However, Y_2_O_3_ was not detected in the XRD results, possibly due to the low content of yttrium in the alloy.

Al has the highest affinity for oxygen except for yttrium and is prone to forming stable Al_2_O_3_ oxides as shown in Figure 15. The grain boundaries are rapid diffusion channels for oxygen, and the internal oxidation of Al is controlled by the inward diffusion of oxygen ions through the external oxide layer and grain boundaries [53]. The content of Cr in GH4169 superalloy is much higher than that of Al, and the self-diffusion coefficient of Al in Al_2_O_3_ is several orders of magnitude smaller than that of Cr and Ti in their oxides [31]. The average diffusion distance of aluminum ions is too short to move through the oxide, and the diffusion coefficient of Al in the alloy is also several orders of magnitude smaller than that of Cr and Ti. This also implies that the growth of Al_2_O_3_ proceeds by inward diffusion of oxygen rather than by outward diffusion of aluminum ions in the oxide. Consequently, Al_2_O_3_ is formed in the innermost layer of the oxide scale [30]. This is consistent with the results of this study.

## 5. Conclusions

In this study, the effect of yttrium on the oxidation behavior of GH4169 Ni-based superalloy and its oxidation mechanism at extreme temperature were studied. The main conclusions are as follows:

(1) The appropriate addition of yttrium can reduce the oxidation weight gain of the alloy, reduce the thickness of the oxide scale and the oxidation rate of GH4169 Ni-based superalloy, and improve the oxidation resistance of the alloy. Excessive addition of yttrium will worsen the high-temperature oxidation resistance of the alloy. The oxidation kinetics curve of alloys with different additions of yttrium at 1000 °C lies between a parabola and a cubic law;
(2) The oxide scale of GH4169 is mainly divided into four layers. The outermost layer (I layer) has a relatively loose structure and is mainly composed of Cr_2_O_3_ and spinel NiCr_2_O_4_. The subouter layer (II layer) consists predominantly of Cr_2_O_3_ oxide with a dense structure; The subinner layer (III layer) comprises mainly Nb-Ti-O phase, composed of TiO_2_ and Nb_2_O_5_. The innermost layer (IV layer) is the inner oxide Al_2_O_3_;
(3) The appropriate addition of yttrium can promote the formation of alloy Cr_2_O_3_ oxide scale, reduce the vacancy defect at the oxide scale/matrix interface, inhibit the formation of interfacial voids, and improve the anti-spallation ability of the alloy and the adhesion of oxide scale.

## Figures and Tables

**Table 1:** Composition of yttrium in the test superalloy (wt.%).

Alloy	Additive Designation	Actual Composition
Y0	-	-
Y1	0.01	0.0052
Y2	0.05	0.04
Y3	0.15	0.11
Y4	0.3	0.25

**Table 2:** Phase chemical composition of the as-cast GH4169 Ni-based superalloys analyzed by EDS.

Region	Alloy	Element (wt.%)							
		Ni	Cr	Fe	Mo	Nb	Ti	C	Y
Matrix	Y0	47.91	16.65	14.86	3.37	5.72	1.03	10.46	-
	Y2	48.96	17.17	15.43	3.16	5.19	0.95	9.11	0.03
	Y4	48.79	17.36	15.91	3.12	4.67	0.91	9.22	0.02
Laves	Y0	31.52	11.04	9.20	5.90	28.79	0.87	12.67	-
	Y2	33.13	9.46	8.50	6.75	29.55	0.87	11.61	0.13
	Y4	32.24	10.81	9.50	6.99	28.85	0.79	10.59	0.23
MC	Y0	1.77	0.64	0.60	2.66	69.05	6.76	18.52	-
	Y2	2.11	1.00	0.83	1.51	71.02	5.84	17.58	0.11
	Y4	1.39	0.81	0.49	1.75	71.05	6.96	17.38	0.17

**Table 3:** EDS results of element distribution in each oxide layer of the alloy in Figure 9.

Alloy	Oxide Layer	Point	Element (wt.%)							
			Ni	Cr	Fe	Nb	Ti	Al	Y	O
Y0	I	A	21.88	26.81	21.7	0.25	0.07	0	-	29.29
	II	B	0.52	62.75	2.77	0.38	0.65	0.03	-	32.9
		C	37.3	28.48	10.08	2.7	1.88	0.39	-	19.17
	III	D	1.86	8.77	0.81	46.97	14.82	0.45	-	26.32
	IV	E	26.6	5.02	10.25	1.29	0.14	30.24	-	26.46
Y2	I	F	3.13	49.68	9.58	0.45	2.61	0.28	-	34.27
	II	G	1.03	60.97	0.41	0.28	1.49	1.08	-	34.74
	III	H	0.43	8.45	0.96	51.6	10.32	0.36	0.76	27.12
	IV	I	24.74	3.68	8.79	0.6	0.52	33.88	-	27.79

**Table 4:** EDS results of element distribution in each oxide layer of the alloy in Figure 10.

Alloy	Oxide Layer	Point	Element (wt.%)							
			Ni	Cr	Fe	Nb	Ti	Al	Y	O
Y0	I	A	24.4	26.29	22.95	0.63	0.24	0.01	-	25.48
	II	B	0.48	63.64	4.51	0.28	0.4	0	-	30.69
		C	0.12	22.69	0.37	38.73	7.21	0.1	-	30.78
	III	D	1.47	7.47	0.07	52.49	10.82	0.13	-	27.55
	IV	E	6.44	6.83	3	0.71	3.21	43.36	-	36.45
Y2	I	F	27.52	32.76	16.76	0.31	0.04	0.29	-	22.32
		G	57.63	4.44	15.57	10.93	1.18	0	0.12	10.13
	II	H	0.75	65.68	1.95	0.58	0.84	0.61	-	29.59
	III	I	0.91	8.31	2.37	49.26	12.85	0.21	0.72	25.37
	IV	J	9.54	4.46	5.21	0.57	0.81	43.22	0	36.19

**Table 5:** Oxidation weight gain reaction index (*n*) and oxidation rate constant (*K_p_*) of GH4169 Ni-based superalloy at 1000 °C.

Alloy	*n*	*K_p_* (mg^n^·cm^−2n^·h^−1^)
Y0	2.43	5.21 × 10^−2^
Y1	2.17	3.70 × 10^−2^
Y2	2.13	3.19 × 10^−2^
Y3	2.27	4.31 × 10^−2^
Y4	2.47	5.76 × 10^−2^

**Table 6:** Gibbs free energy of oxide formation at 1000 °C (kJ·mol^−1^).

NiO	Cr_2_O_3_	TiO_2_	Al_2_O_3_	Y_2_O_3_	Fe_2_O_3_	Nb_2_O_5_
−250.0	−535.4	−714.2	−847.0	−1024.8	−327.6	−539.7

## Data Availability

Data are contained within the article.
